# Supplementary material for: Genomic Survey of Genes Encoding Major Intrinsic Proteins (MIPs) and Their Response to Arsenite Stress in Pepper (Capsicum annum)
Source: Plants (Basel). 2025 May 14;14(10):1475. doi: 10.3390/plants14101475 (PMC12114714; doi:10.3390/plants14101475)

Supplementary Materials:

Supplementary Table S1; Ka/Ks analysis of MIP genes in pepper plant

CA genes	AT genes	Ks	Ka	Ka/Ks
**CATIP1-12**	ATTIP1-1	1.71	0.15	0.09
**CATIP1-10**	ATTIP1-3	1.29	0.13	0.10
**CATIP2-3**	ATTIP2-1	1.65	0.12	0.07
**CATIP2-4**	ATTIP2-3	1.55	0.15	0.10
**CATIP2-4**	ATTIP2-2	1.73	0.12	0.07
**CATIP2-4**	ATTIP2-1	1.78	0.15	0.08
**CAPIP1-2**	ATPIP1-4	1.75	0.13	0.08
**CAPIP1-2**	ATPIP1-2	1.45	0.15	0.11
**CAPIP1-2**	ATPIP1-3	1.61	0.08	0.05
**CAPIP1-2**	ATPIP1-5	1.26	0.10	0.08
**CAPIP1-2**	ATPIP1-1	1.47	0.12	0.08
**CAPIP1-8**	ATPIP1-4	1.43	0.14	0.10
**CAPIP1-8**	ATPIP1-2	1.50	0.15	0.10
**CAPIP1-8**	ATPIP1-5	1.63	0.10	0.06
**CAPIP1-8**	ATPIP1-3	2.27	0.09	0.04
**CAPIP1-8**	ATPIP1-1	1.72	0.11	0.07
**CAPIP1-1**	ATPIP1-4	3.57	0.13	0.04
**CAPIP1-1**	ATPIP1-2	1.60	0.16	0.10
**CAPIP1-1**	ATPIP1-5	3.03	0.11	0.03
**CAPIP1-1**	ATPIP1-3	1.79	0.10	0.06
**CAPIP1-1**	ATPIP1-1	1.45	0.12	0.08
**CAPIP1-5**	ATPIP1-4	1.52	0.13	0.08
**CAPIP1-5**	ATPIP1-5	2.85	0.09	0.03
**CAPIP1-5**	ATPIP1-2	1.70	0.14	0.09
**CAPIP1-5**	ATPIP1-3	1.87	0.11	0.06
**CAPIP1-5**	ATPIP1-1	1.86	0.11	0.06
**CAPIP1-4**	ATPIP1-4	2.71	0.15	0.06
**CAPIP1-4**	ATPIP1-5	1.54	0.11	0.07
**CAPIP1-4**	ATPIP1-2	1.74	0.18	0.11
**CAPIP1-4**	ATPIP1-3	1.12	0.10	0.09
**CAPIP1-4**	ATPIP1-1	1.94	0.12	0.06
**CAPIP2-5**	ATPIP2-7	1.29	0.10	0.08
**CAPIP2-5**	ATPIP2-8	1.03	0.12	0.12
**CAPIP2-2**	ATPIP2-7	2.38	0.09	0.04
**CAPIP2-2**	ATPIP2-8	2.13	0.10	0.05
**CAPIP2-1**	ATPIP2-2	2.30	0.17	0.08
**CAPIP2-1**	ATPIP2-4	2.28	0.16	0.07
**CAPIP2-1**	ATPIP2-6	3.15	0.20	0.06
**CAPIP2-1**	ATPIP2-3	2.62	0.20	0.08
**CAPIP2-1**	ATPIP2-8	2.63	0.20	0.07
**CAPIP2-1**	ATPIP2-5	2.08	0.20	0.10
**CAPIP2-1**	ATPIP2-7	2.13	0.19	0.09
**CAPIP2-1**	ATPIP2-1	1.45	0.19	0.13
**CAPIP2-6**	ATPIP2-2	4.04	0.13	0.03
**CAPIP2-6**	ATPIP2-3	4.05	0.13	0.03
**CAPIP2-6**	ATPIP2-4	1.77	0.15	0.09
**CAPIP2-6**	ATPIP2-1	2.07	0.14	0.07
**CAPIP2-4**	ATPIP2-2	1.74	0.14	0.08
**CAPIP2-4**	ATPIP2-4	2.80	0.13	0.05
**CAPIP2-4**	ATPIP2-1	2.38	0.13	0.05
**CAPIP2-4**	ATPIP2-8	2.42	0.17	0.07
**CAPIP2-3**	ATPIP2-2	2.59	0.14	0.05
**CAPIP2-3**	ATPIP2-4	1.91	0.13	0.07
**CAPIP2-3**	ATPIP2-5	2.11	0.14	0.07
**CAPIP2-3**	ATPIP2-1	2.18	0.12	0.06
**CAPIP2-3**	ATPIP2-8	1.82	0.17	0.10

**CANIP5-1**	ATNIP5-1	2.51	0.13	0.05

Note: Ka (Non-synonymous)/Ks (Synonymous) analysis were performed by"One Step MCScanx" and "Simple Ka/Ks Calculator"function.

Supplementary Table S2; Primer sequences of MIP genes used for qRT-PCR in pepper. Primer Quest Tool was used to design primers for MIP gene family (http://www.idtdna.com/PrimerQuest/Home/Index?Display=AdvancedParams).

Name	Sequence	Name	Sequence
**CAPIP1-8F**	ACCACCAGCACCTTTGTT	CANIP1-2F	GCTGGAGGAGCTAATGGAAATA
**CAPIP1-8R**	CACCACCAAGTCTCTGATAAGG	CANIP1-2R	GCAAGTGGCAAAGGCAATAG
**CAPIP2-7F**	ACAGAGCAGCAATAGCAGAG	CATIP2-3F	AATTCGTCACCGGAGGATTG
**CAPIP2-7R**	GTGGCAGAGAAGACAACATAGA	CATIP2-3R	ACTGCAGGCCCAAATGAA
**CATIP1-14F**	CATTTGTGGGTGGACACATTAC	CANIP6-1F	CTCTAGTGGACTCGCTGTAATG
**CATIP1-14R**	GGAGTTCCTCATCGGTGTTT	CANIP6-1R	GCGTGATTGTTGCTGTCTTC
**CATIP1-13F**	AGAGGCTAGACATCCTGATACC	CATIP3-1F	GCTGGTGAAGGTTCTGTTCT
**CATIP1-13R**	GCTACTGCTGTGAGAGAGAATG	CATIP3-1R	CAATGGCAAGAGGTGCAATG
**CATIP4-1F**	TGCCATGGCTGCCAATAA	CATIP5-1F	CAACACTCTGTCACTCCCAAT
**CATIP4-1R**	GCAGCAACAGAAGCCAATAAC	CATIP5-1R	GAAGGGTCCAATCCAGTACAC
**CATIP1-12F**	GCGGTTGGTGCTAACATTTC	CASIP1-1F	TATTGGGTGGTGCTGGTTTC
**CATIP1-12R**	CGGTTCATGGGTTTGGTTAATG	CASIP1-1R	GTCAAGACACCCTCTGCAATAG
**CAPIP2-6F**	CACCTCCAGCACCACTTATT	CANIP4-3F	GGAGGTCATAGGGACGTATTTC
**CAPIP2-6R**	TAGCTCCACCACCGTACTTA	CANIP4-3R	CCAGCTAGGGTTCCAACTATT
**CAPIP2-5F**	ATCCTCCTCCAGCTCTCTTT	CANIP1-1F	GTGAACGCAGACAAAGGAATG
**CAPIP2-5R**	CTAGCAGGGTTGATACCAGTTC	CANIP1-1R	AGACCAGCAAGTTCTCCAATAG
**CAPIP2-4F**	GTATCGTTGGTTCGGGCTATTA	CANIP2-1F	ACCGTTGGGCATGTTTCT
**CAPIP2-4R**	GCTCCAGCCCTCAAGATAAA	CANIP2-1R	GCCCAGCAAGGATAGAGTTAAT
**CAPIP2-3F**	CCCTTGATTGACCCTGAAGAA	CATIP1-7F	CTGATTGCAGCCACGATAAAC
**CAPIP2-3R**	CACCACCACCATACCTGAAATA	CATIP1-7R	GAGTTGGTCCCACAGATGATAA
**CAPIP2-2F**	GCAGTGACATTTGGGTTGTTC	CANIP4-2F	CACGTATCGGGTGGTCATTT
**CAPIP2-2R**	GGGTTGATACCAGTTCCAGTAAT	CANIP4-2R	CAAGTTGTCCTACCGCTCTATC
**CAPIP1-7F**	GGCTGCTGCTAACGGAAATA	CANIP5-1F	GTTCAAGTGCCAGCCTATGT
**CAPIP1-7R**	GCCAGAAGCCCAACAATAGA	CANIP5-1R	GACTGTAGCTCCAACTGCTATG
**CAPIP1-6F**	GGCCTTGGTGCTGAGATTAT	CAPIP1-2F	CACCAGCAGCACCTTTATTTG
**CAPIP1-6R**	ACGCATGTTCTCCGTTGTAG	CAPIP1-2R	CACCGCCAAGTCTCTGATATG
**CAPIP1-5F**	GGCAACCATTAGGGACTTCA	CATIP1-6F	GAAATCTGAGAGAGGCTACCAAG
**CAPIP1-5R**	CTGAGATACCAGCAGTGCAATA	CATIP1-6R	AGTAATGTGTCCACCCACAAG
**CATIP1-11F**	CACCTTTGGCGCCTTTATTG	CATIP1-5F	TTCCCTGCTGAAGGTTGTG
**CATIP1-11R**	CAGTGGACCCAGTGGTTATT	CATIP1-5R	CCGCAACAGATCCCAGTAAT
**CATIP1-10F**	GAAGGGTGACTTGGGAATCAT	CANIP3-1F	GTGGGAGGTTCTATATTGGCTAC
**CATIP1-10R**	AGCTGCTCATGAGTGTTCTG	CANIP3-1R	CTCCTGCAACAGCTCCTATAAT
**CATIP1-9F**	CTGGCATGGCCTTCAGTAA	CATIP1-4F	ACTGCTCTCTTCCTCCTCAA
**CATIP1-9R**	AGCACACAAGCCACAATAGA	CATIP1-4R	CGAATGGACCGAACCAGTAAA
**CATIP1-8F**	GCTGTTACTTTCGGTGCTTTC	CATIP1-3F	AGAGGCTACCCATCCTGATAC
**CATIP1-8R**	ATCTCCTCCACTTGGGATTTG	CATIP1-3R	TGAGCGTTTCGAGAGCTAATG
**CANIP2-2F**	CCGCTATTACCGCTACTTCTAC	CATIP1-2F	ACTGCTCTCTTCCTCCTCAA
**CANIP2-2R**	GAGTGGATCCTACGACTTGAAC	CATIP1-2R	CGAATGGACCGAACCAGTAAA
**CATIP3-2F**	AAGGTATGCGTTTGGGAGAG	CATIP2-2F	CACCATTGGATTGGCTGTTG

**CATIP3-2R**	GGACCACCTACAAGGACATTAG	CATIP2-2R	TCTGCTGCAGTGGCATAAA
**CATIP2-4F**	GGGTCATTGAAGGCCTACTT	CAPIP1-1F	ACTTGGTGGTGGTGCTAATG
**CATIP2-4R**	TTTCCACCTACAGCCAATCC	CAPIP1-1R	GCTGCATCGGTGTTGTAGAT
**CAPIP2-1F**	TCATGGCTCCTGGACATAAC	CATIP1-1F	CTCCACGAAGCGGGTTTATT
**CAPIP2-1R**	GGAGGATGAACTGATGGTAGAC	CATIP1-1R	CATGGTGTTTCTCCACCTCTAAG
**CAPIP1-4F**	GGATGTGAGATTGGGTGCTAAC	CANIP4-1F	GCCTTTGCACTTCACCTAATG
**CAPIP1-4R**	CCATGCGGGTTCCTTGTTATAG	CANIP4-1R	CAGTGGCAACACCAGAAATG
**CAPIP1-3F**	CTGGGCATTCCTGACTTCTT	CATIP2-1F	GTTGCTTGCTACCTCCTCAA
**CAPIP1-3R**	GAAACACCTAGCCGGATTCA	CATIP2-1R	AGTGGTCCAACCCAGTAAATC
**Actin-F**	AGGGATGGGTCAAAAGGATGC		
**Actin-R**	GAGACAACACCGCCTGAATAGC		

Supplementary Figure S1. Pepper growth stage used for experiment. As(III) 0.5 and 1 mM were applied with control (without As(III)). Data was recorded for leaf Chlorophyll content and Nitrogen content using SPAD 502 (Minolta Camera, Co., Osaka, Japan) after 48 hrs of treatment. Root length was measured with ruler. Samples were collected liquid nitrogen for RNA extraction to assess root and leaf response to different concentrations of As(III) using qRT-PCR. Scale bar is 50 mm for each.

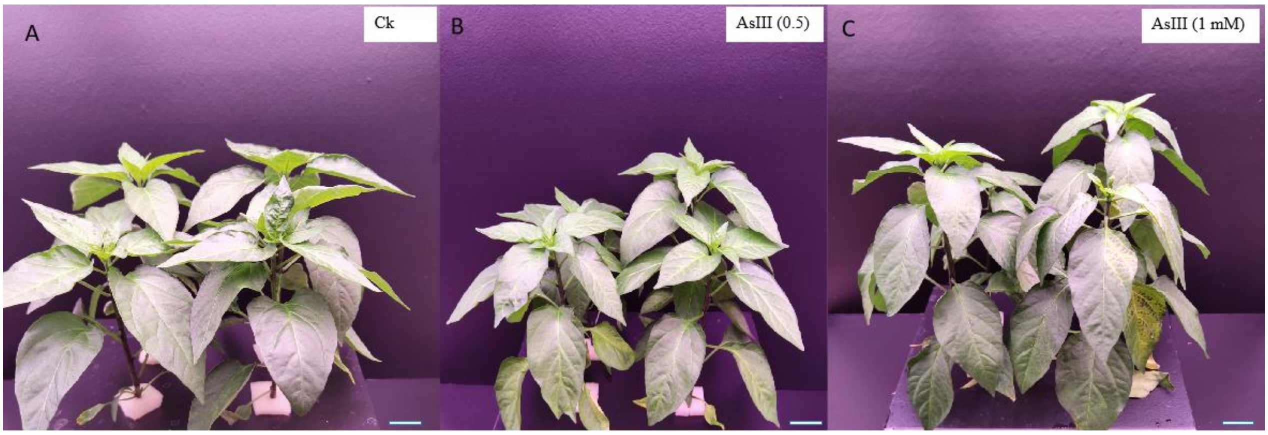

Supplement: Supplementary file 1 [file plants-14-01475-s001.zip › plants-3563946-supplementary.pdf]
